# Interfering Residues Narrow the Spectrum of MLV Restriction by Human TRIM5α

**DOI:** 10.1371/journal.ppat.0030200

**Published:** 2007-12-28

**Authors:** Pierre V Maillard, Séverine Reynard, Fatima Serhan, Priscilla Turelli, Didier Trono

**Affiliations:** Global Health Institute, School of Life Sciences, “Frontiers in Genetics” National Center for Competence in Research, Ecole Polytechnique Fédérale de Lausanne (EPFL), Lausanne, Switzerland; Institute for Research in Biomedicine, Switzerland

## Abstract

TRIM5α is a restriction factor that limits infection of human cells by so-called N- but not B- or NB-tropic strains of murine leukemia virus (MLV). Here, we performed a mutation-based functional analysis of TRIM5α-mediated MLV restriction. Our results reveal that changes at tyrosine^336^ of human TRIM5α, within the variable region 1 of its C-terminal PRYSPRY domain, can expand its activity to B-MLV and to the NB-tropic Moloney MLV. Conversely, we demonstrate that the escape of MLV from restriction by wild-type or mutant forms of huTRIM5α can be achieved through interdependent changes at positions 82, 109, 110, and 117 of the viral capsid. Together, our results support a model in which TRIM5α-mediated retroviral restriction results from the direct binding of the antiviral PRYSPRY domain to the viral capsid, and can be prevented by interferences exerted by critical residues on either one of these two partners.

## Author Summary

Mammalian cells are endowed with intrinsic lines of defence against retroviruses, which notably contribute to limiting the cross-species transmission of these pathogens. TRIM5α is one such restriction factor, which acts by recognizing the capsid of incoming retroviruses through its C-terminal PRYSPRY domain. Human TRIM5α potently blocks the so-called N-tropic murine leukemia virus (MLV), but is ineffective against the closely related B-tropic and Moloney strains. In this study, we demonstrate that substitution of a single amino acid in the PRYSPRY domain of this protein expands its antiviral activity to these other MLV strains. Conversely, we show that protection of MLV from this restriction is governed by the negative influence of specific residues at a few critical positions of the retroviral capsid. These results support the model of a direct interaction between TRIM5α and retroviral capsids, shedding light on an important arm of innate antiretroviral immunity.

## Introduction

Retroelements constitute important evolutionary forces for the genome of higher organisms, yet their uncontrolled spread, whether from endogenous loci or within the context of retroviral infections, can cause diseases such as cancer, autoimmunity and immunodeficiency, including AIDS. Correspondingly, a variety of host-encoded activities limit this process, behaving as the arms of a line of defence commonly called intrinsic immunity, which notably contributes to restricting the cross-species transmission of retroviruses [1].

The product of the Friend virus susceptibility 1 (*Fv1*) gene, which shares similarity with the *gag* region of an endogenous retrovirus, conditions the susceptibility of various mouse strains to murine leukemia virus (MLV) [2,3]. N-tropic and B-tropic MLV strains replicate in Swiss/NIH and in Balb/c mice, respectively, reflecting the presence of either the *n* or the *b* allele of *Fv1* in the genome of these animals. A critical determinant of the differential sensitivity of MLV strains to Fv1 lies in amino acid 110 of the viral capsid (CA), which is an arginine in the prototypic N-tropic MLV and a glutamate in its B-tropic counterpart [4,5]. Moloney MLV (Mo-MLV) harbors an alanine at this position and escapes both Fv1^n^ and Fv1^b^, hence is termed NB-tropic.

N-MLV is restricted too in some non-murine mammalian cells, including of human origin, which do not encode Fv1. Blockade is in these cases mediated by TRIM5α, a member of the tripartite motif (TRIM) family of proteins [6–10]. TRIM5α also prevents the cross-species transmission of primate lentiviruses. The orthologues present in Old World monkeys, including macaque rhesus, restrict human immunodeficiency virus type 1 (HIV-1) and N-MLV, while those from New World monkeys, tend to restrict simian immunodeficiency virus of macaques (SIV_mac_) and for some species N-MLV but not HIV-1 [11–13]. Human TRIM5α (huTRIM5α) blocks N-MLV, but is only weakly active against SIV_mac_ and HIV-1 [6–9,11,14].

All TRIM proteins contain a RING, a B-Box and a coiled-coil region, which together constitute the so-called RBCC domain [10,15,16]. TRIM5α further harbors at its C-terminus the PRYSPRY or B30.2 domain, responsible for the viral capsid-specific capture of restricted viruses [17–19]. Sequence alignments of the PRYSPRY domains of various primate TRIM5α and other related TRIM proteins reveal 4 variable regions (V1, V2, V3 and V4), predicted to constitute surface-exposed loops based on the structure of the homologous domain of related proteins [12,20–23]. While V1, V2 and V3 were all found to contribute to the antiviral specificity of TRIM5α orthologues [11], V1 was shown to play a most critical role in this process. Within this loop, removing a positive charge at position 332 or substituting residues 335 to 340 by an eight amino acid rhesus sequence confers huTRIM5α with the ability to restrict HIV-1 [11,24,25]. Conversely, introducing residues 335–340 of huTRIM5α at the corresponding locus of rhesus monkey TRIM5α (rhTRIM5α) enhances the N-MLV blocking activity of the simian protein [26].

The present study was designed to define further how TRIM5α recognizes retroviral capsids. Our results indicate that, if huTRIM5α efficiently restricts only N-MLV and not B-MLV or Mo-MLV, this is due to the negative influence of a key residue in V1. Conversely, MLV is capable of avoiding restriction via the interdependent influences of a cluster of amino acids exposed at the surface of its capsid.

## Results

### A Single Amino Acid Substitution in huTRIM5α PRYSPRY Domain Extends Its Activity to B- and Mo-MLV

**Figure 1:**
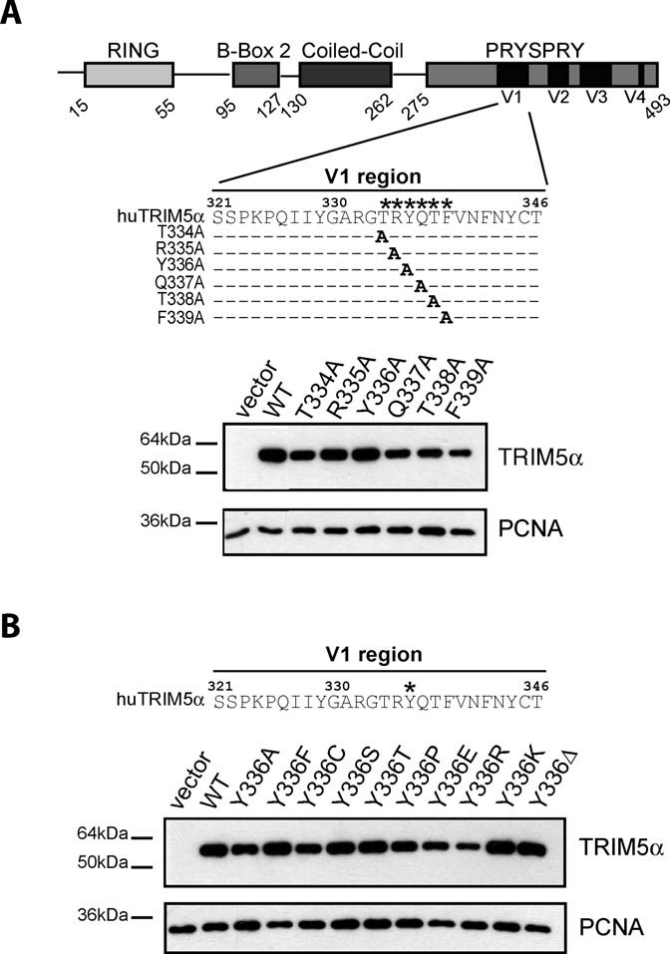
Stable Expression of Wild-Type or PRYSPRY V1 Mutant Forms of huTRIM5α (A) Schematic representation of the domains present in huTRIM5α. Numbers refer to the amino acid position. V1 through V4 designate the four variable regions found in the PRYSPRY domain. The V1 amino acid sequence is shown below, with mutated amino acids (334 to 339) indicated with an asterisk (*). Below is a western blot analysis of extracts from MDTF cells stably transduced with retroviral vectors expressing HA-tagged versions of these huTRIM5α derivatives, using HA (top) and PCNA (bottom)-specific antibodies. (B) Same analysis, with derivatives carrying the indicated amino acid substitutions or a deletion (Δ) at position 336.

**Figure 2:**
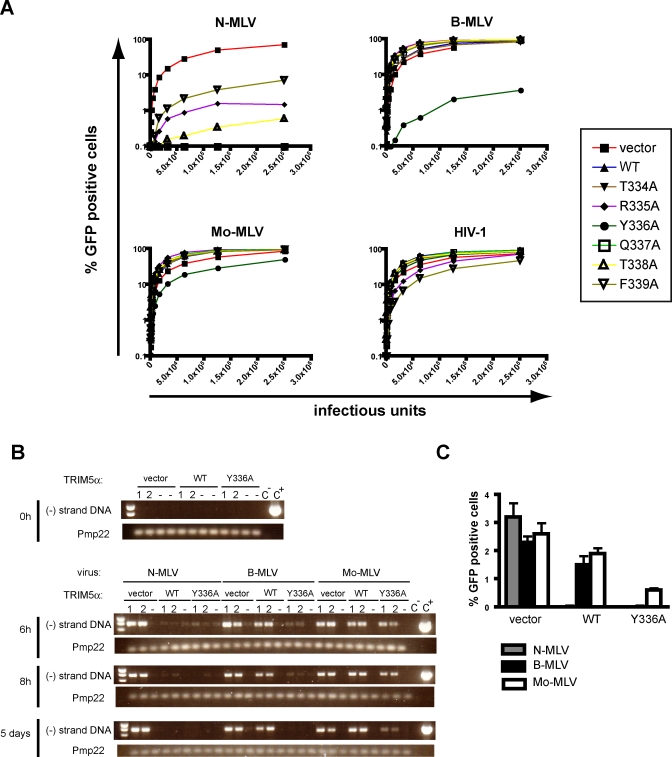
HuTRIM5α_Y_336_A_ Potently Blocks B-MLV at an Early Stage of Infection (A) MDTF cells expressing wild-type (WT) or indicated mutant forms of huTRIM5α, or transduced with a control vector (vector), were challenged with serial 2-fold dilutions of MLV-based or HIV-1-based GFP vectors initially titered on permissive MDTF cells. Infections were scored at 48 hours post-infection by FACS. Curves are representatives of at least two independent experiments, and phenotype observed with huTRIM5α_Y_336_A_ was confirmed in three independently obtained stable cell lines. (B) Cells expressing wild-type or Y^336^A mutated huTRIM5α, or control cells (vector), were challenged with equal doses of N-, B- or Mo-MLV. Cellular DNA extractions were performed before (0 h) or 6 h, 8 h or 5 days post-infection. Intermediate minus strand DNA reverse transcription products were amplified by PCR. For each cell line, transduction was performed in triplicate in the absence (1, 2) or presence (-) of azidothymidine (AZT), a reverse transcription inhibitor. Water (C^−^) and the plasmid pCNCG (C^+^) were used as negative and positive controls for the PCR. The mouse peripheral myelin protein (Pmp22) gene was used as a loading control. (C) In parallel of the experiment described in (B), the percentage of GFP-positive cells was determined 5 days post-infection by flow cytometry.

In order to characterize the interaction between huTRIM5α and its viral targets, we introduced amino acid changes in the central V1 region of its PRYSPRY domain. Cell lines stably expressing the resulting mutants were generated by retroviral vector-mediated transduction of permissive *Fv1*-null Mus dunni tail fibroblasts (MDTFs) (Figure 1). A first series of MDTF derivatives expressing huTRIM5α mutants carrying single alanine substitutions at positions 334 to 339 were challenged with MLV- or HIV-derived green fluorescent protein (GFP)-expressing vectors, scoring infection by fluorescence-activated cell sorting (FACS) analysis (Figures 1A and 2A). All mutants conserved the ability to restrict N-MLV, albeit at a slightly reduced efficiency for some (e.g., F^339^A). None could block HIV-1, except for F^339^A that was lowly active. In contrast, replacement of tyrosine^336^ by alanine yielded a mutant capable of efficiently blocking B-MLV and, to a small extent, Mo-MLV.

We examined the step of the B-MLV replicative cycle targeted by this expanded-spectrum huTRIM5α mutant. Several reports have demonstrated that N-MLV blockade by huTRIM5α occurs at an early post-entry stage, before reverse transcription [7,13,27]. In contrast, the only restriction activity so far identified against B-MLV is mediated by Fv1^n^, which allows viral DNA synthesis to proceed but inhibits viral nuclear import [28,29]. We thus infected MDTF cell lines expressing either wild-type or Y^336^A huTRIM5α, or control cells, with equal doses of N-, B- or Mo-MLV vectors and monitored the accumulation of reverse transcription products by PCR, using primers that amplified elongated minus-strand DNA (Figure 2B). All three vectors yielded readily detectable reverse transcripts in control cells at 6 hours post-infection. Consistent with previous studies, N-MLV DNA levels were significantly reduced in the presence of wild-type huTRIM5α, whereas B-MLV escaped this effect. In contrast, both N- and B-MLV exhibited strikingly reduced amounts of reverse transcripts in cells expressing huTRIM5α_Y_336_A_. With Mo-MLV, a slight decrease in viral DNA was noted in cells expressing this mutant at 8 hours post-infection, compared with control or wild-type huTRIM5α-expressing cells. This inhibition was more obvious when the analysis was repeated at 5 days post-infection, that is, upon scoring the ultimate proviral load of the cells, which correlated with the results of the FACS analyses performed at the same time (Figure 2B and 2C). Altogether, these data indicate that wild-type and huTRIM5α_Y_336_A_ similarly act before the completion of reverse transcription.

### Y^336^ Exerts Negative Influence to Limit the Spectrum of huTRIM5α Antiviral Activity

**Figure 3:**
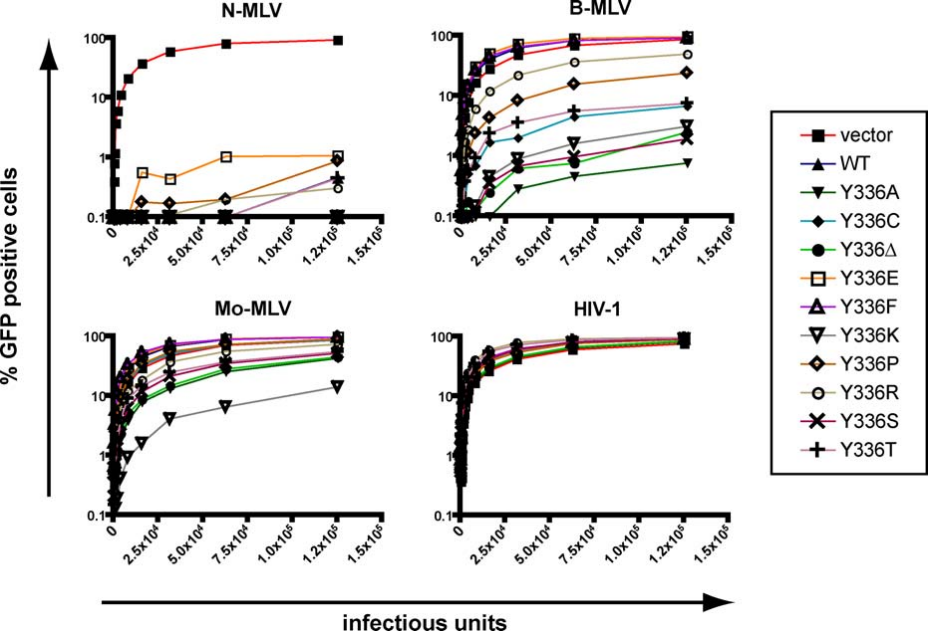
Differential Restriction Spectrum of huTRIM5α Y^336^ Mutants MDTF cells expressing indicated huTRIM5α derivatives, as documented in Figure 1B, were challenged with serial dilutions of MLV- and HIV-based GFP vectors, and analyzed by flow cytometry 2 days later. The resulting infectivity curves are representatives of at least two independent experiments.

To explore further the modalities by which the Y^336^A mutation renders huTRIM5α active against B-MLV, we generated MDTF cell lines expressing huTRIM5α derivatives with other amino acid substitutions or with a deletion at this position (Figure 1B). N-MLV restriction was unaffected by any of these changes. In contrast, variable degrees of B-MLV restriction were observed (Figure 3). Introduction of other small amino acids besides alanine (threonine, serine, cysteine and, to a lesser extent, proline), or removal of tyrosine^336^, also conferred a gain-of-function phenotype to huTRIM5α. One mutant, huTRIM5α_Y_336_K_, even acquired the ability to block Mo-MLV with a good efficiency (Figure 3). This did not simply reflect the presence of a positive charge at this position, because the Y^336^R mutation rendered huTRIM5α only weakly active against B-MLV and even less so against Mo-MLV, even though it might be due to its poor expression level. Notably, replacing tyrosine^336^ with glutamate or phenylalanine did not broaden huTRIM5α restriction beyond N-MLV. Moreover, none of the mutants acquired the ability to block HIV-1.

### B-MLV Restriction Seems Incompatible with HIV-1 Blockade

**Figure 4:**
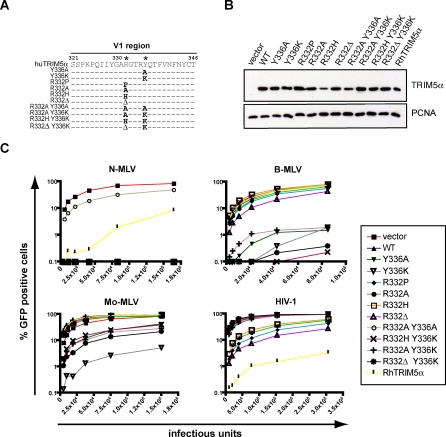
B-MLV and HIV Restriction Are Mutually Exclusive (A) Single and double point mutations (*) were introduced at positions 332 and 336 in the V1 region of huTRIM5α as illustrated. (B) MDTF cell lines stably expressing these mutants were engineered by retroviral transduction, and analyzed by western blot as described in Figure 1. (C) These cells were challenged with 2-fold dilutions of either N-MLV, B-MLV, Mo-MLV or HIV-derived GFP-expressing vectors, and infections were scored by flow cytometry as described in Figure 2. Plots are representative of at least two independent experiments.

It was previously demonstrated that a single amino acid substitution or deletion at position 332 of huTRIM5α provides this molecule with the ability to block HIV-1 infection [24,25,30]. We thus asked whether a molecule carrying changes at both positions 332 and 336 restricts not only N- and B-MLV, but also HIV-1. For this, we generated MDTF cell lines expressing a series of huTRIM5α double mutants, using various combinations of substitutions and deletions previously noted as broadening the spectrum of activity of the protein towards either B-MLV (this work) or HIV-1 [24,25,30] (Figure 4A). Double mutants that included an alanine substitution at position 336 were either poorly expressed (R^332^H/Y^336^A, data not shown) or expressed but without antiviral activity even against N-MLV (R^332^A/Y^336^A, Figure 4B and 4C). All other double mutants were stably expressed, and exhibited the MLV restriction patterns expected from the residue present at position 336 (Figure 4B and 4C). Single mutants with arginine to proline, histidine, alanine or a deletion at position 332 significantly restricted HIV-1, albeit it less efficiently than rhTRIM5α. However, none of the double mutants was effective against this virus. Furthermore, substitutions at position 332 somewhat reduced the ability of huTRIM5α_Y_336_K_ to restrict Mo-MLV (Figure 4C). It thus appears that changes underlying the acquisition of B- and Mo-MLV restriction ability preclude further extension of the activity of huTRIM5α to HIV-1.

### Context-Limited Influence of Residue 110 of the MLV Capsid on Susceptibility to huTRIM5α-Mediated Restriction

**Figure 5:**
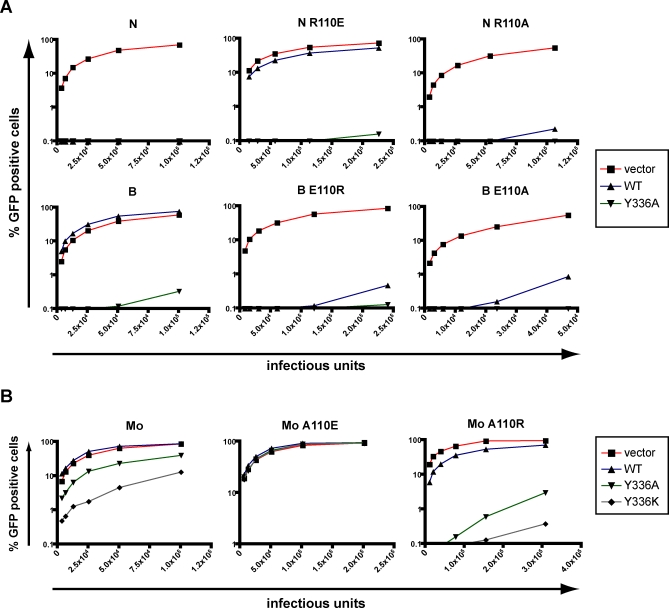
Context-Specific Influence of MLV Capsid Residue 110 MDTF cells stably expressing wild-type or mutant versions of huTRIM5α, or containing a control vector, were challenged with N-, B- or Mo-MLV-derived vectors packaged with wild-type or CA110-mutated capsids, as indicated. Infections were scored as described in Figure 2.

The effective blockade of both N- and B-MLV by several huTRIM5α mutants strongly suggested that the “canonical” amino acid 110 of the MLV capsid, the importance of which for Fv1^n^ and Fv1^b^ sensitivity has been extensively demonstrated [4,5,27], did not play an essential role in the case of TRIM5α. The N- and B-MLV packaging constructs sequence used in the present study encode for viral capsids that differ in only three residues (CA109, CA110 and CA159) [27,31,32]. Exchanging the residue present in either virus at position 110, through reciprocal arginine-glutamate exchanges, sufficed to confer wild-type huTRIM5α susceptibility or resistance to N- and B-MLV. However, both mutants were potently blocked by huTRIM5α_Y_336_A_, which also restricted either N- or B-MLV modified to contain an alanine at this position (Figure 5A). These data confirmed that TRIM5α can be altered to act in a CA110-independent manner. However, in the context of Mo-MLV, this capsid residue plays a pivotal role. Mo-MLV could indeed escape all forms of TRIM5α-mediated blockade when alanine^110^ of its capsid was changed to glutamate. Inversely, when arginine was introduced instead, Mo-MLV restriction by huTRIM5α_Y_336_K_ and huTRIM5α_Y_336_A_ was strengthened, and the virus became slightly sensitive to wild-type TRIM5α, as recently noted [33] (Figure 5B).

### CA82, CA109, CA110, and CA117 Interdependently Affect MLV Sensitivity to huTRIM5α

**Figure 6:**
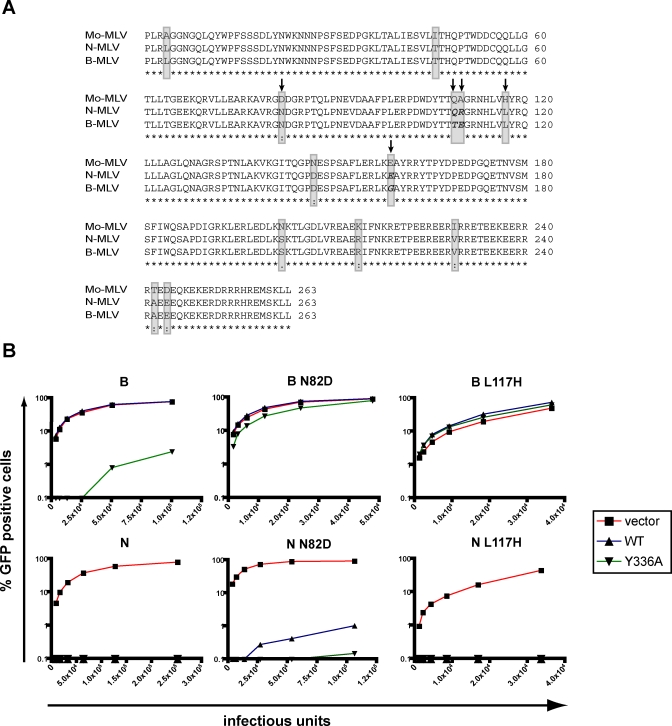
MLV CA82 and CA117 Can Interfere with TRIM5α-Mediated Restriction (A) Amino acids sequence alignments of Mo-, N- and B-MLV capsids. Residues that differ between Mo-MLV and N-MLV and/or B-MLV are highlighted in grey. The residues in italics at positions 109, 110 and 159 represent the only amino acids differences between N- and B-MLV capsids. Positions targeted by site-directed mutagenesis in the present study are indicated by an arrow. (B) Infectivity assays with indicated cell lines and vectors, performed as described in Figure 2. Residues at positions 82 and 117 exert different influences whether in an N- or B-MLV context.

**Figure 7:**
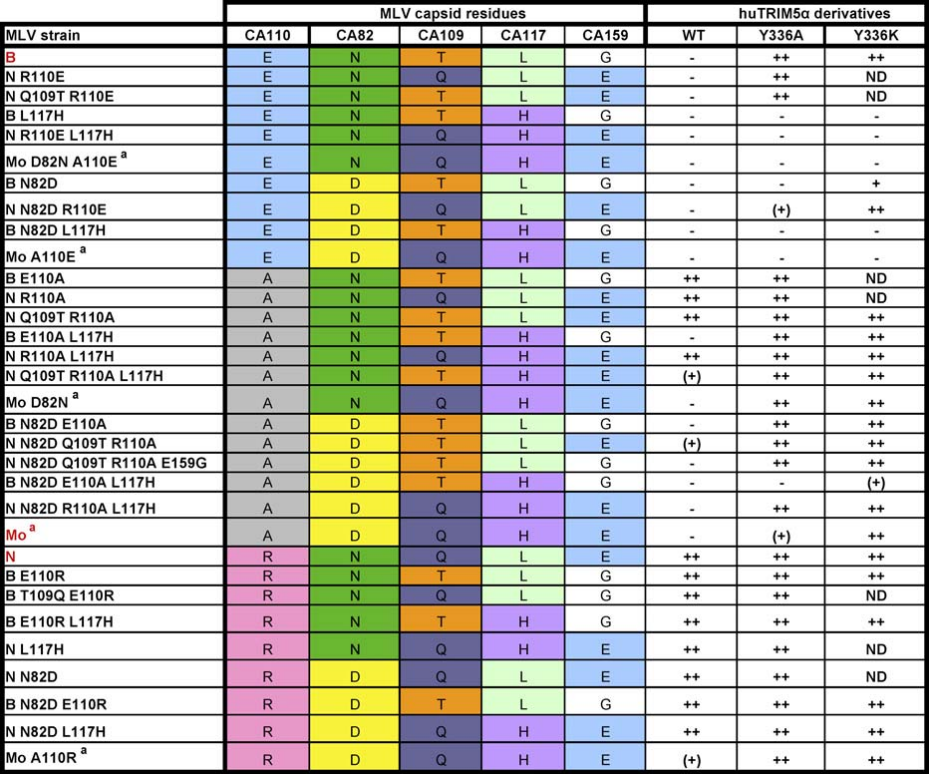
Susceptibility to huTRIM5α-Mediated Restriction of Various MLV Derivatives ^a^In addition to CA110, CA82, CA109, CA117, and CA159, Moloney MLV capsid differs from N- and B-MLV CA at eight other positions (CA4, CA46, CA147, CA202, CA214, CA229, CA242, CA244). Fold restriction: -, less than 2; (+), 2 to 5; +, 5 to 10; ++, more than 10 (calculated as described in Materials and Methods); ND: not done. The one-letter abbreviation of amino acids was used to designate residues present at CA110, CA82, CA109, CA117, and CA159 (A: alanine, D: aspartate, E: glutamate, G: glycine, H: histidine, L: leucine, N: asparagine, Q: glutamine, R: arginine, T: threonine).

We then sought to define which other capsid residues influence MLV susceptibility to huTRIM5α-mediated blockade. For this, we focused on amino acids in N- or B-MLV that differ from Mo-MLV, and on positions previously demonstrated to influence restriction by huTRIM5α and/or Fv1 (Figure 6A) [34,35]. We found that single point mutations at positions 82 (N to D) or 117 (L to H) of capsid allowed B-MLV to escape completely huTRIM5α_Y_336_A_ restriction (Figure 6B). Nevertheless, the influence of these two mutations was context-dependent, because when introduced in N-MLV they relieved neither wild-type nor huTRIMa_Y_336_A_-mediated restriction (Figure 6B). Furthermore, the newly introduced residues are those naturally present in Mo-MLV, which is blocked by huTRIM5α_Y_336_K_ (Figures 3 and 5B). The testing of a high number of additional single and combined mutants confirmed that MLV susceptibility to TRIM5α-mediated restriction is dictated by the interdependent influences of capsid residues 82, 109, 110 and 117 with a minor modulation by residue 159 (Figure 7).

## Discussion

It is suspected, albeit not yet formally demonstrated, that TRIM5α-mediated retroviral restriction proceeds through the direct binding of the antiviral PRYSPRY domain to the capsid of incoming viruses [13,17,18,24,27]. The present study, which demonstrates that the consequences of mutations in the huTRIM5α PRYSPRY V1 can be counterbalanced by changes in the MLV capsid, and vice versa, lends strong credence to such a model.

This work stands out by its identification of a residue, tyrosine^336^ of huTRIM5α, which limits the spectrum of MLV targets of this antiviral to the sole N-tropic MLV. A number of amino acid substitutions at this position, as well as a deletion of this residue, confer huTRIM5α with the additional ability to block B-MLV, and introduction of a lysine even expands restriction to Mo-MLV. Understanding fully the mechanism of this gain of function would require a determination of the tri-dimensional structure of the TRIM5α-capsid complex. In its absence, the crystal structures of the PRYSPRY domain of related proteins, PRYSPRY-19q13.4.1, GUSTAVUS and TRIM21, suggests that the V1 region of huTRIM5α could form a protruding loop with tyrosine^336^ situated underneath and in direct contact (at a less than 4 Å distance) with residues in the V2 loop, which could limit the conformational flexibility of V1 [20–22]. However, the PRYSPRY V1 loops of TRIM5α and these other proteins differ in length, precluding firm analogy [23]. Alternatively, tyrosine^336^ may prevent the docking of V1 into its putative capsid-binding site by steric hindrance. This would be consistent with the finding that changes that most effectively broaden the spectrum of action of huTRIM5α are the removal of this tyrosine or its substitution by small amino acids. However, the observation that Y^336^K further expands the restriction spectrum of huTRIM5α not only to B- but also to Mo-MLV suggests that this model may be overly simplistic. Arginine^332^ of huTRIM5α was similarly found to interfere with ability of huTRIM5α to restrict HIV-1 and SIV_mac_ [30]. In this case too, distinct amino acid changes differentially affected the strength with which either one of these two viruses was inhibited, suggesting that both positive and negative influences are at play. As well, our failure to generate a TRIM5α variant capable of blocking both N- and B-MLV on the one hand and HIV-1 on the other hand, by combining mutations at positions 332 and 336, points to more complex influences within V1 itself. *cis*-acting interferences have also been noted in Fv1, where the C-terminal part of Fv1^b^ was shown to prevent this factor from blocking B-MLV, and where substitution of lysine^358^ of Fv1^n^ by alanine could extend the restriction spectrum of this antiviral to N-MLV [36].

On the viral capsid side, our study indicates that at least four positions (CA82, CA109, CA110 and CA117) interdependently condition MLV susceptibility to huTRIM5α, whether in its wild-type or mutant forms. The influence of each of these four residues varies according to both the virus involved and the sequence of the TRIM5α PRYSPRY V1 region. Here, all viruses tested escaped wild-type huTRIM5α if they harbored a glutamic acid at position 110 of capsid. As such, E^110^ dominantly interfere with restriction. However, a recent study demonstrated that wild-type huTRIM5α could efficiently block an MLV retroviral vector packaged with a capsid derived from a primate endogenous retrovirus (PtERV) carrying a glutamic acid at this position [37]. As well, we found here that the protective effect of E^110^ could be abrogated by substitutions of Y^336^ in huTRIM5α, in which case CA82 and CA117 became determinant. Indeed, with N- and B-MLV-derived viruses, an aspartate at CA82 additionally allowed escape from TRIM5α_Y_336_A_, and a histidine at CA117 from TRIM5α_Y_336_A_ and TRIM5α_Y_336_K_. When CA110 was occupied by an arginine, the picture was completely reversed, as this residue dominantly potentiates susceptibility. Finally, with an alanine at CA110, H^117^ and D^82^ induced escape from wild-type TRIM5α, albeit in a CA109-dependent fashion, yet viruses remained sensitive to Y^336^-mutated forms of the restriction factor.

A picture is thus emerging from these data, whereby CA110 plays the role of primary determinant of restriction, with CA82, CA109 and CA117 acting as secondary modulators in a V1-conditioned fashion. However, the restriction pattern obtained with derivatives of Mo-MLV, which differs from the N- and B-MLV strains used here at nine CA positions besides these four, does not fully fit with this model, indicating its modulation by at least some of these other CA residues. Notably, a recent study demonstrated the importance of CA214 in potentiating Fv1^n^-mediated restriction of Mo-MLV only when CA110 was occupied by a glutamate [33].

MLV CA82, CA109, CA110 and CA117 were also demonstrated to exert combinatorial influences on Fv1-mediated restriction [35]. In spite of this parallel, sequences leading to resistance or susceptibility to Fv1 and huTRIM5α are not identical. For instance, whereas Fv1^b^ and huTRIM5α both potently restrict CA_E_110_A_ B-MLV, an additional N^82^D mutation allows escape from huTRIM5α (this work) but not from Fv1^b^ [35]. Also, huTRIM5α_Y_336_A_-mediated blockade of B-MLV is relieved by change at CA117, which was previously shown not to affect restriction by Fv1^n^ [35].

**Figure 8:**
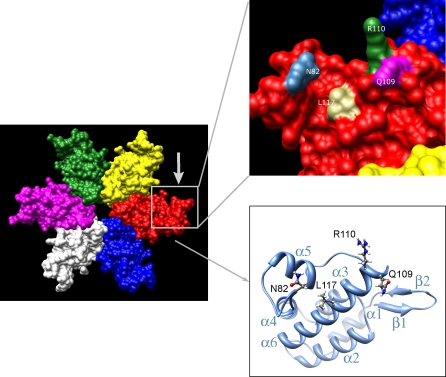
Highlighting of Functionally Critical Positions on the Structure of the MLV Capsid Visualization of the previously defined structure of the N-terminal domain of the N-MLV capsid in its hexameric state, each monomer being given a different color. Secondary structures in one monomer with the six α helices (α1–6) and two β-hairpins (β1 and β2) are represented in the lower right panel. The four residues at positions 82, 109, 110 and 117 are shown with their respective side chains. Atoms in the side chains are colored in grey for carbon, white for hydrogen, red for oxygen and blue for nitrogen. In the upper right panel, the molecular surface of the area framed in the red-colored monomer is enlarged and viewed in the orientation indicated by the arrow. Surfaces of the four residues at positions 82, 109, 110, and 117 are colored.

The structure of the amino-terminal part of the N-MLV capsid in its hexameric state was resolved at a 2.5 Å resolution [38] (Figure 8). A monomer consists of two-stranded β-hairpins followed by six α helices. Interestingly, CA82, CA109, CA110 and CA117 are situated at the edge of a cavity formed by helices 4 to 6 (Figure 8). CA82 sits between helix 4 and 5 at the top of this pocket, across from CA109 and 110 on helix 6. CA117 is further down along the helix 6 side of the cavity. At least two scenarios can thus be envisioned for the binding of huTRIM5α to the MLV capsid. First, it might rely on the sum of individual interactions between TRIM5α residues, for instance in the PRYSPRY V1 loop, and capsid amino acids including 82, 109, 110 and 117. The non-essential nature of any of these four capsid positions for susceptibility or resistance to TRIM5α argues against this model, even though it is conceivable that the abrogation of some of these interactions might be compensated by the strengthening of others. In a second scenario, the TRIM5α-binding site would be located deeper in the pocket. This part of the protein is constituted by residues that are highly conserved, hence most probably play essential structural functions prohibiting mutation [38]. Escape could then be achieved by mounting obstacles to TRIM5α penetration into this pocket through changes at the more flexible yet critically placed CA82, CA109, CA110 and CA117 residues. By analogy, it is interesting to note that the cyclophilin-binding loop of the HIV-1 capsid, which has been postulated to interfere with the blockade of this virus by wild-type huTRIM5α, hangs over a very similar pocket formed by helices 4 to 7 of the structurally homologous lentiviral capsid [38,39]. As such, this loop, whether bound to or modified by cyclophilin A (CypA), could function as a lid to prevent huTRIM5α from accessing its HIV-1 CA binding site. However, recent data, which indicate that the positive effects of CypA binding to CA on HIV-1 replication do not depend upon the presence of huTRIM5α suggest that a strict parallel cannot be established between restriction of MLV and HIV by the cellular antiviral [40–42].

## Materials and Methods

### Cell lines and culture.

Mus dunni tail fibroblasts (MDTFs) and human embryonic kidney 293T cells (HEK 293T) were purchased from the American Type Culture Collection (ATCC). All cell lines were cultivated in Dulbecco's modified Eagle medium supplemented with 10% fetal calf serum, 2 mM glutamine and antibiotics (100 U/ml penicillin, 100 mg/ml streptomycin).

### Plasmids.

MLV-based particles were produced using packaging constructs containing Moloney MLV (pCIGPB), N- and B-tropic MLV CA (pCIG3-N and pCIG3-B) kindly provided by O. Danos and J. Stoye, respectively [32]. The GFP-encoding vector construct for all MLV reporter viruses was pCNCG kindly provided by R. Zufferey. Lentiviruses-based vectors were produced with the packaging construct psPAX2 (Figures 2A and 3) or pR8.74 (Figure 4) and the vector pWPTS-GFP (Figures 2A and 3) or pRRLsin PGK GFP (Figure 4). The *env* construct for all viral productions was pMD2G plasmid expressing vesicular stomatitis virus G protein. Many plasmids used are distributed by Addgene (http://www.addgene.org/).

The MLV plasmid encoding human TRIM5α was a kind gift of J. Sodroski and was already described [13]. The amino acid coding sequence of human TRIM5α with a C-terminal epitope derived from influenza virus hemagglutinin (HA) was inserted in pLPCX MLV vector construct (Clontech) allowing for puromycin selection of transduced cells. Site-directed mutagenesis on pLPCX-huTRIM5α-HA, pCIG3-N, pCIG3-B and pCIGPB was performed with the XL QuickChange mutagenesis kit from Stratagene. Primers used are listed in Table S1. Proper site-directed mutagenesis was checked by sequencing reactions.

### Viral production.

All vector productions were performed by CaPO_4_-mediated transient co-transfection of the retroviral vector, *gag-pol* and *env* encoding constructs (http://tronolab.epfl.ch/; with some minor adjustments). Briefly, subconfluent HEK 293T cells were co-transfected with 21.5 μg of vectors, 14.6 μg packaging constructs and 7.9 μg *env* constructs in a 15-cm plate. Cells were washed 16 hours post-transfection and supernatants were harvested 12, 24 and 36 hours later. Recombinant retroviral vectors containing supernatants were centrifugated, filtrated, and in some cases were concentrated by ultracentrifugation. Titrations were performed on *Fv1*-null MDTF cells.

### Engineering of stable wild-type and mutant TRIM5α-expressing cell line.

MLV-based retroviral vectors encoding wild-type or point mutants of human TRIM5α were produced using the pLPCX-derived plasmids as described above. Viral supernatants containing recombinant retroviral vectors were added on 5 × 10^4^ MDTF cells. Forty-eight hours post-transduction, cells were expanded and selection for stably transduced cells was performed by adding puromycin (Sigma) at a concentration of 5 μg/ml. Cells were maintained continuously in the presence of puromycin.

To evaluate TRIM5α expression level, total proteins were extracted in a radioimmune precipitation assay buffer (phosphate-buffered-saline (PBS) with 1% NP-40, 0.5% sodium deoxycholate and 0.1% SDS) supplemented with protease inhibitor cocktail (Calbiochem). Equal amounts of protein were resolved on a Tris-glycine SDS-Polyacrylamide gel followed by western blot. HA-tagged proteins were detected using peroxydase-conjugated rat monoclonal antibody (clone 3F10, Roche). Proliferating cell nuclear antigen (PCNA) was used as a protein loading control and was detected using a mouse monoclonal antibody (clone PC10, Calbiochem) followed by a secondary sheep anti-mouse antibody conjugated to horseradish peroxidase.

### Reverse transcript detection.

MDTF cells stably expressing wild-type huTRIM5α, huTRIM5α_Y_336_A_ or stably transduced with the empty pLPCX construct as a control were seeded at 2.5 × 10^4^ in a 24-well plate. N-, B- and Mo-MLV viral stocks encoding GFP were treated with DNAse I (20 μg/ml) in the presence of MgCl_2_ (10 mM) for 30 minutes at 37°C. Cells were then transduced at an equal low multiplicity of infection. For all time points and for each cell line, a PCR negative control with azidothymidine (62.5 μM, Calbiochem) pre-treated cells was included. Cells were then harvested before transduction and 6 or 8 hours post-transduction. DNA was then extracted using the DNAeasy Tissue extraction kit from Quiagen. To detect the presence of provirus, cells were also collected 5 days post-transduction and processed for DNA extraction and FACS analysis. PCR reactions were performed using 5 μl of DNA extract. PCR amplified a region from the neomycin resistance gene (forward primer: 5' GCGTTGGCTACCCGTGATATTG 3') to the cytomegalovirus promoter (reverse primer: 5' TGGGCTATGAACTAATGACC 3') present in the intermediate reverse transcript resulting from RNA expressed by pCNCG. Mus musculus peripheral myelin protein (Pmp22, NM_008885) was used as a normalization gene (forward primer: 5' TTCGTCAGTCCCACAGTTTTCTC 3', reverse primer: 5' ACTCGCTAGTCCCAA GGGTCTA 3').

### Infection with GFP reporter vectors and calculation of the fold restriction.

MDTF stable cell lines were seeded at 2.5 × 10^4^ and transduced 24 hours later with 2-fold serial dilutions of GFP reporter vectors. Cells were harvested 48 hours post-transductions and fixed in 1% formaldehyde-containing PBS. The percentage of GFP-positive cells was determined by flow cytometry using the Beckton Dickinson FACScan or the multi-well plate reader Beckman Coulter Cell lab Quanta Flow Cytometer. Results were analysed with FlowJo 8.1.1 software.

To calculate the fold restriction of the different MLV capsid mutants by huTRIM5α derivatives, a ratio was performed between the percentage of GFP-positive cells in the absence (cells stably transduced with the empty vector) and presence of huTRIM5α derivatives (cells stably expressing wild-type or mutants huTRIM5α). Ratios were calculated with each dose of GFP vector from at least two independent infections, and the average of these ratios was used for the semi-quantitative scoring given in Figure 7.

### Molecular imaging.

The resolved structure of the N-terminal domain of N-MLV capsid in its hexameric state ([38]; PDB: 1U7K) was visualized using the UCSF Chimera software as described [43].

## Supporting Information

Table S1 Sequences of the Primers Used for the Site-Directed Mutagenesis Modified nucleotides to generate the specified amino acid substitution are highlighted in bold and are underlined. Site where nucleotides were removed to create amino acid deletion are indicated with “//”.
(29 KB XLS)

### Accession Numbers

The National Center for Biotechnology Information (http://www.ncbi.nlm.nih.gov/) accession numbers for the proteins discussed in this paper are human TRIM5α (AY625000), peripheral myelin protein Pmp22 (NM_008885), and Rhesus TRIM5α (AY523632).

## Abbreviations

AIDS: acquired immunodeficiency syndrome
AZT: azidothymidine
B-MLV: B-tropic MLV
CA: capsid
FACS: fluorescence-activated cell sorting
Fv1: Friend virus susceptibility 1
GFP: green fluorescent protein
HA: hemagglutinin epitope
Hu: human
HIV-1: human immunodeficiency virus type 1
MDTF: *Mus dunni* tail fibroblast
Mo-MLV: Moloney MLV
MLV: murine leukemia virus
N-MLV: N-tropic MLV
PBS: phosphate-buffered saline
PCNA: proliferating cell nuclear antigen
Rh: rhesus
SDS: sodium dodecyl sulfate
SIV_mac_: simian immunodeficiency virus of macaques
TRIM5α: tripartite motif 5α
V: variable region
